# Structure of the polymerase ε holoenzyme and atomic model of the leading strand replisome

**DOI:** 10.1038/s41467-020-16910-5

**Published:** 2020-06-22

**Authors:** Zuanning Yuan, Roxana Georgescu, Grant D. Schauer, Michael E. O’Donnell, Huilin Li

**Affiliations:** 1 Structural Biology Program, Van Andel Institute, Grand Rapids, MI USA; 2 Howard Hughes Medical Institute, Chevy Chase, MD USA; 3 DNA Replication Laboratory, The Rockefeller University, New York, NY USA; 4 Present Address: Department of Biochemistry and Molecular Biology, Colorado State University, Fort Collins, CO 80523 USA

**Keywords:** DNA, Enzyme mechanisms, DNA synthesis, Cryoelectron microscopy

## Abstract

The eukaryotic leading strand DNA polymerase (Pol) ε contains 4 subunits, Pol2, Dpb2, Dpb3 and Dpb4. Pol2 is a fusion of two B-family Pols; the N-terminal Pol module is catalytic and the C-terminal Pol module is non-catalytic. Despite extensive efforts, there is no atomic structure for Pol ε holoenzyme, critical to understanding how DNA synthesis is coordinated with unwinding and the DNA path through the CMG helicase-Pol ε-PCNA clamp. We show here a 3.5-Å cryo-EM structure of yeast Pol ε revealing that the Dpb3–Dpb4 subunits bridge the two DNA Pol modules of Pol2, holding them rigid. This information enabled an atomic model of the leading strand replisome. Interestingly, the model suggests that an OB fold in Dbp2 directs leading ssDNA from CMG to the Pol ε active site. These results complete the DNA path from entry of parental DNA into CMG to exit of daughter DNA from PCNA.

## Abstract (web summary)

DNA polymerase epsilon (Pol ε) is responsible for leading strand synthesis during DNA replication. Here the authors use Cryo-EM to describe the architecture of the Pol ε holoenzyme and to provide an atomic model for the leading strand replisome.

## Introduction

**Fig. 1:**
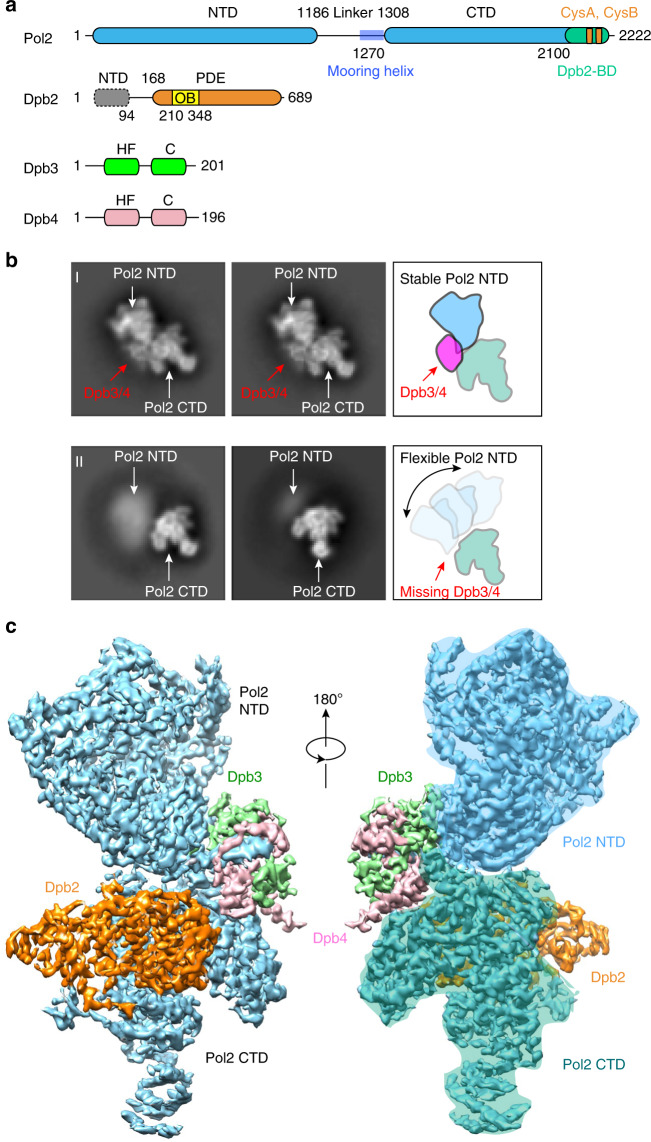
Cryo-EM structure of the *S. cerevisiae* leading strand Pol ε. **a** Domain architecture of the four subunits of the holoenzyme: Pol2 contains two polymerase domains, the catalytic NTD and the non-catalytic CTD. There is a Dpb2-binding domain (Dpb2-BD) in the CTD that further contains two Cys motifs (CysA and CysB). Dbp2 has an OB domain and a calcineurin-like PDE domain. Dpb3 and Dbp4 each contain a histone fold domain (HF) and a C-terminal region (C). **b** 2D class averages of Pol ε showing the rigid state (I) and flexible state (II). **c** 3D map in front and back views, with each subunit shown in a distinct color.

Chromosome replication in eukaryotes is performed by three different B-family DNA polymerases (Pol), Pol ε, Pol δ, and Pol α-primase^1–4^. Pol ε performs bulk leading strand synthesis while Pol δ acts on the lagging strand. Pol α-primase contains both RNA primase and DNA polymerase activity and functions to generate hybrid RNA-DNA primers to initiate DNA synthesis by Pol ε and Pol δ. Pol ε is the largest of the replicative DNA polymerases and contains four subunits (Fig. 1a)^5,6^. The Pol2 subunit harbors the catalytic DNA polymerase and proofreading 3′–5′ exonuclease in the N-terminal half. The three accessory subunits include the essential Dpb2 and two small nonessential Dpb3 and Dpb4 subunits. The small Dpb3 and Dpb4 subunits each adopt histone folds that form a tight Dpb3–4 complex^7^. Pol ε physically associates with the replicative CMG (Cdc45, Mcm2-7, and GINS) helicase to assemble a molecular machine termed the leading strand replisome that couples continuous DNA unwinding with high fidelity and processive DNA synthesis^8–10^. Pol2 contains two DNA polymerase modules covalently linked in a 2222-residue long polypeptide chain; the catalytic polymerization and proofreading nuclease action are contained in the N-terminal (NTD) module of Pol2 while the C-terminal (CTD) module of Pol2 encodes a non-catalytic DNA polymerase that likely serves a structural role^11^. Genetic studies in *Saccharomyces cerevisiae* (S.c.) show that the inactive polymerase module of Pol2 is essential, while the catalytic N-terminal module of Pol2 is not essential, although cell growth is quite compromised^12,13^. The Dpb2 subunit is also essential^14^, and studies indicate that it functions with the CTD inactive polymerase module of Pol2 in assisting initiation factors in the formation of CMG helicase at origins^15–17^. Genetic studies reveal that the Dpb3 and Dpb4 histone fold subunits are not essential^18,19^, but are required for preservation of epigenetic information during replication^20,21^.

Structures of individual subunits and domains of S.c. Pol2 have previously been determined. The active NTD of Pol2 bound to primed DNA is solved to 2.5 Å resolution^22^ and the inactive CTD of Pol2 is determined to 4.5–7 Å resolution^15^. The structure of Dpb2 and crystal structure of the histone fold subunits Dpb3–Dpb4 are also previously reported^7,15,23^. However, the arrangement of these subunits and domains within the Pol ε holoenzyme is unknown due to the inability to trap a rigid form of the holoenzyme. Thus, the location of Dpb3–4 complex in the Pol ε holoenzyme and the orientation of the Pol2 NTD and CTD in the holoenzyme are not known. Furthermore, the Dpb3–4 complex is demonstrated to bind double-strand DNA and enhance the processivity of Pol ε ^7,24,25^, but exactly how this occurs is not understood due to the lack of knowledge about the orientation of Dpb3–4 relative to the Pol2 subunit.

This report determines the structure of the S.c. Pol ε holoenzyme, revealing the juxtaposition of each of the subunits. A most interesting finding is that the active and inactive polymerase modules of Pol2 are spatially separate and are held together by the Dpb3–Dpb4 histone fold subunits. Importantly, the Pol ε structure has enabled us to build a pseudo atomic model of the leading strand replisome, revealing the orthogonal path of the parental DNA entering CMG and the nascent daughter DNA exiting from PCNA, and how the leading single-strand DNA is directed by the Dpb2 OB domain from the CMG helicase to the Pol ε active site.

## Results and discussion

### The Pol ε holoenzyme is held by Dpb3–4 into a rigid state

In electron micrographs, Pol ε is a flexible two-lobed structure, with the Pol2 NTD in lobe 1, the Pol2 CTD and Dpb2 in lobe 2, and the Dpb3–4 position unknown. Our 2D classification of a large cryo-EM dataset of Pol ε revealed the full Pol ε holoenzyme in a rigid form and reveals that Dpb3–4 binds between lobe 1 and lobe 2, holding them rigid. (Fig. 1b, top row). We also observed particles that displayed the previously observed flexibility in which the image classes only resolved lobe 2 (Fig. 1b, bottom row). Specifically, we observed averaged class images with all subunits, including both Pol2 NTD and CTD domains, Dpb2, and Dpb3–4 complex (lobes 1 and 2), but also class averages with Pol2 CTD and Dpb2 (lobe 1) and blurry Pol2 NTD lobe and missing or blurry Dpb3–4. Previous cryo-EM studies of Pol ε (and Pol ε–CMG complex) have only visualized the lobe 1 state of Pol ε^10,15,26^. Thus, we were surprised to observe class averages in which both lobes 1 and 2 had well-defined structural features (Fig. 1a), showing that Pol ε holoenzyme can exist in a rigid form. We presume that the plunge-freezing process needed to make cryo-EM grids often disrupts the Pol ε holoenzyme, although it remains possible that Pol ε has two functional forms, rigid and flexible. The possible function of a flexible form of Pol ε holoenzyme, assuming it exists in the cell, will be considered below.

### The structure of the rigid state Pol ε holoenzyme

Through large-scale data collection including recording data at a tilted angle of 30° and 3D classification selecting the rigid particles, we obtained a cryo-EM 3D map at 3.5 Å of the Pol ε holoenzyme (Fig. 1c, Supplementary Figs. 1–3, and Supplementary Table 1). At this resolution one could observe side chains at numerous positions in the 3D map (Supplementary Fig. 4). Atomic model building was facilitated by the large side chain densities, as well as the previously determined structures of each component of Pol ε. This is the first time that the structure of a eukaryotic leading strand DNA polymerase holoenzyme has been determined to atomic resolution, revealing the position and orientation of each of the four subunits.

**Fig. 2:**
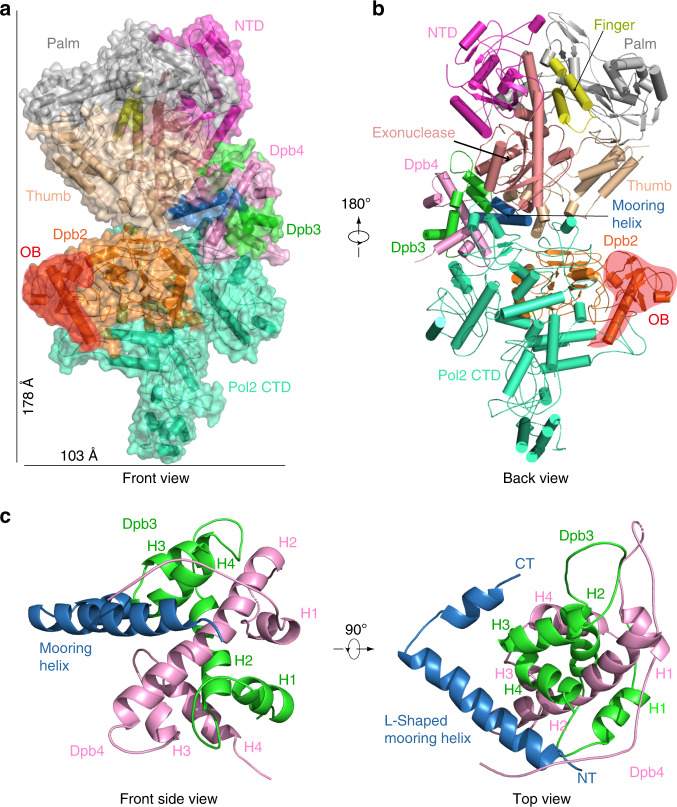
Atomic model of the Pol ε. **a** Front view of Pol ε in cartoon and surface views. **b** Back view of Pol ε in cartoon view only. In **a**, **b** each subunit and each subdomain of Pol2 catalytic NTD are individually colored. The Dpb2 OB domain is shadowed red to highlight its position in front of the template DNA entrance in the middle of Pol2 NTD. **c** Enlarged view of the Dpb3–Dpb4 site, highlighting the mooring helix that anchors and braces Dpb3 and Dpb4. The four helices of the histone fold in Dpb3–4 are labeled H1 through H4. The N-terminal and C-terminus of Dpb4 and the L-shaped mooring helix are labeled by NT and CT, respectively.

Lobe 1 of the holoenzyme contains the Pol2 catalytic domain with an N-terminal subdomain (31–281), an exonuclease (282–527), a palm (528–950), a finger (769–833), and a thumb (951–1186) domain that are organized into a toroid (Fig. 2a). The Pol2 NTD structure is an open circle compared with the crystal structure of the DNA template/primer (T/P)-bound Pol2 NTD (Supplementary Fig. 5a, b)^22^. The difference in our apo structure and the ternary complex reveals a 27° tilt of the finger domain. These observations indicate that the native state of the Pol2 apo-enzyme forms a gapped circle, and that DNA/dNTP binding induces the finger domain to clamp down to form the conformation that completely encircles the DNA.

Lobe 2 contains Pol2 CTD and Dpb2. Dpb2 is the B subunit conserved in eukaryotic Pols α, δ, and ε^27,28^ and contains an N-terminal largely helical domain, an OB domain and an inactivated calcineurin-like phosphoesterase domain (PDE) (Figs. 1a and 2b)^29^. In our structure, the PDE domain (residues 168–209 and 349–689) and the embedded OB domain (residue 210–348) are well ordered, but the N-terminal helical domain (residues 8–94) is disordered. The Dpb2 subunit forms intimate contacts to the Pol2 CTD via the PDE but has few contacts with the Pol2 NTD or with the Dpb3–4ubcomplex. The crystal structure of the B subunit (p59) in complex with the p261 C-terminal Zn-coordinating fragment (p261c CysA and CysB) of human Pol ε was previously solved to 2.35 Å^29^. The human crystal structure superimposes well with the corresponding region in the yeast Pol ε (Supplementary Fig. 5c). Furthermore, the entire lobe 2 (Pol2 CTD and Dpb2) was recently determined by cryo-EM to 5 Å resolution^15^ revealing that the polymerase fold has a wide-open jaw that is partially blocked by one of the two Cys motifs (CysA). It was unknown if the jaw-blocking CysA motif observed in the Pol2 NTD-truncated structure moves out in the holoenzyme. In our 3.5 Å structure of the holoenzyme, we found the overall structure of Pol2 CTD is similar and the CysA motif remains in place to block the jaw, providing further support that the Pol2 CTD has neither polymerase activity nor DNA-binding activity.

The Dpb3–4 heterodimer is known to have a histone fold, but its position within the Pol ε has been unknown. Interestingly, we found that Dpb3–4 is wedged in the middle of the holoenzyme between lobes 1 and 2, and that there is a solvent exposed and positively charged surface in the Dpb3–4 (Supplementary Fig. 6). Dpb3–4 enhances the processivity of Pol ε holoenzyme and can directly bind DNA in vitro^7,24,25^. It is currently unclear if the positive patch accounts for the reported DNA-binding activity of Dpb3–4, and if so, whether it binds DNA in the context of the replisome. Importantly, we found that the overall architecture of the Pol ε observed in the rigid state is consistent with our previous cross-linking mass spectrometry of the yeast Pol ε in complex with the CMG helicase^10^ (Supplementary Fig. 7), indicating the physiological relevance of the rigid state.

### A mooring helix in the Pol2 NTD–CTD linker anchors Dpb3–4

The 112-residue NTD–CTD linker (Thr-1186–Ser-1308) is widely assumed to be disordered accounting for the flexible association between the two Pol ε lobes. Interestingly, the last one third (Val-1270–Ser-1308; 38 residues) of the linker forms a long L-shaped α-helix in our structure (Fig. 2c and Supplementary Movie 1). This helix appears to be critically important, as it interacts with the Pol2 NTD and recruits Dpb3–4 into the Pol ε holoenzyme. We refer to this α-helix as the mooring helix. The mooring helix underlies the observed rigid state of Pol ε as it positions the Dpb3–4 complex into the weakest middle region of Pol ε and forms intimate contacts to both Pol ε lobes, thereby fixing their orientation relative to one another. This mooring helix was not observed in the Pol2 CTD–Dpb2 subcomplex structure^15^ and likely forms a structured helix only upon interaction with Dpb3–4. The recruited Dpb3–4 then acts as a bridge to buttress the catalytically active NTD and catalytically inactive CTD polymerase modules of Pol2, holding them rigid. The fact that the rigid Pol ε state conforms with our earlier reported in vitro cross-linking/mass spectrometry results further supports our conclusion that the rigid class of particles populate the solution phase state of Pol ε^10^.

**Fig. 3:**
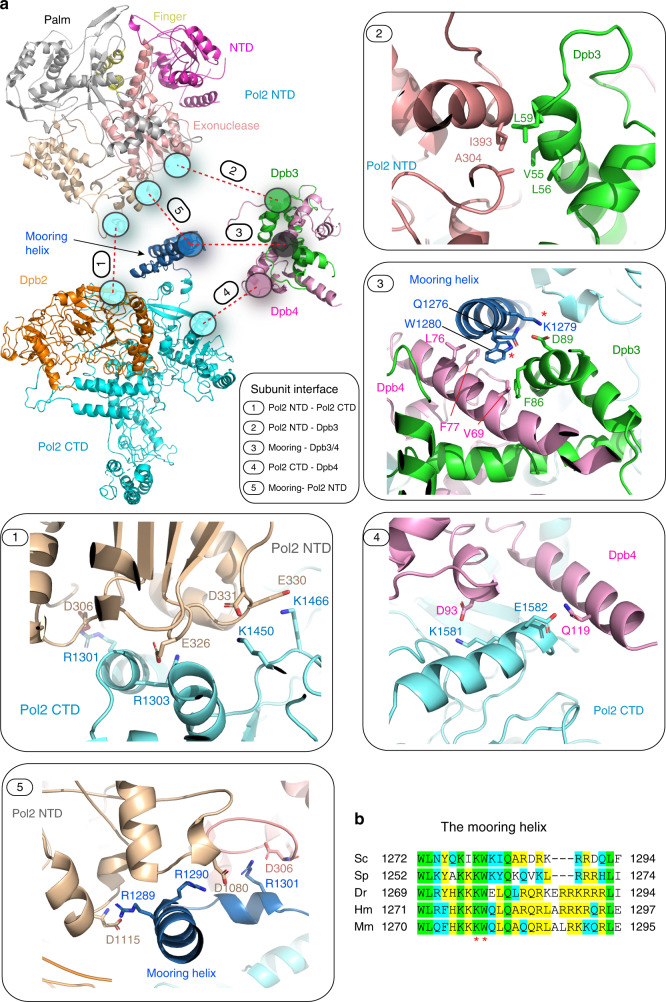
Domain–domain interactions within Pol ε. **a** Pull-apart of the structure. Each circle represents the contact region between two domains. The oval with a number indicates the interaction that is enlarged and shown in the numbered enlarged boxes. 1: between Pol2 NTD and CTD; 2: between Pol2 NTD and Dpb3; 3: between mooring helix and Dpb3–4; 4: between Dpb3 and Dpb4; and 5: between mooring helix and Pol2 NTD. **b** Sequence alignment of the mooring helix in Sc (*S. cerevisiae*), Sp (*S. pombe*), Dr (*D. reos*), Hs (*H. sapien*), Mm (*M. muscarus*). The red asterisks indicate conserved residues involved in interactions with Dpb3–4.

Figure 3a illustrates the detailed connections among: (1) Pol2 NTD and CTD (panel 1), (2) Pol2 NTD and Dpb3 (panel 2), (3) the mooring helix and Dpb3–4 (panel 3), (4) the Pol2 CTD and Dpb4 (panel 4), and (5) the mooring helix and Pol2 NTD (panel 5). The interface between the Pol2 NTD and CTD lacks hydrophobic buried residues and consists mainly of four salt bridges wherein the NTD contains four negatively charged residues and the CTD contains four positive charged residues. These electrostatic salt bridges are unlikely to have sufficient energy to hold the two Pol2 domains in a fixed position. Panels 2 and 4 show the connections between Dpb3–4 and the Pol2 NTD and CTD, respectively. Dpb3 binds the NTD of Pol2, and Dpb4 binds the CTD of Pol2. While the Pol2 NTD forms a 988 A^2^ buried hydrophobic interface with Dpb3 (panel 2), the Pol2 CTD–Dpb4 interface appears less robust involving mainly salt bridges. This may seem curious, considering the established ability of Dpb3–4 (and Dpb2) to form a stable isolable complex with the CTD of Pol2^12,13^. However, this can be explained by the extensive interface of 2307 A^2^ between the Pol2 mooring helix and the Dpb3–4 complex (Fig. 3a, panel 3). Hence, we conclude that it is the mooring helix that anchors Dpb3–4 to Pol2.

Given the importance of the mooring helix to the rigid state of Pol ε holoenzyme, we examined its conservation among eukaryotes (Fig. 3b). Many of the hydrophobic residues are conserved (W1272, L1273, W180, I183, Q184, and L1293). Some of the salt bridges of this interface are also conserved. A previous biochemical study of the human Pol ε described the interaction between p12–p17 (yeast Dpb3–Dpb4) and the NTD–CTD linker region of human p261 (yeast Pol2; 1211–1303)^30^. Hence, we expect the mooring helix will be a broadly conserved structural feature of Pol ε holoenzyme among eukaryotes. We found that a cluster of three charged residues in the Pol2 mooring helix R1285, D1286, and R1287, corresponding to human enzyme R1284, Q1285, and R1286, contained six missense mutations in cancer patients (Supplementary Fig. 8).

### Atomic modeling of the leading strand replisome

Pol ε is known to be physically associated with the replicative helicase CMG to form the leading strand replisome^8,9,31^. Our earlier studies of the solution structure of Pol ε holoenzyme utilized cross-linking mass spectrometry (CX-MS) to confirm subunit orientation within a negative stain EM 3D model of Pol ε holoenzyme bound to CMG^10^. While the negative stain EM did not detect the complete Pol ε holoenzyme, the CX-MS data showed inter-subunit cross-linking data that is fully consistent with the ordered rigid state of Pol ε holoenzyme shown in this report (Supplementary Fig. 7).

**Fig. 4:**
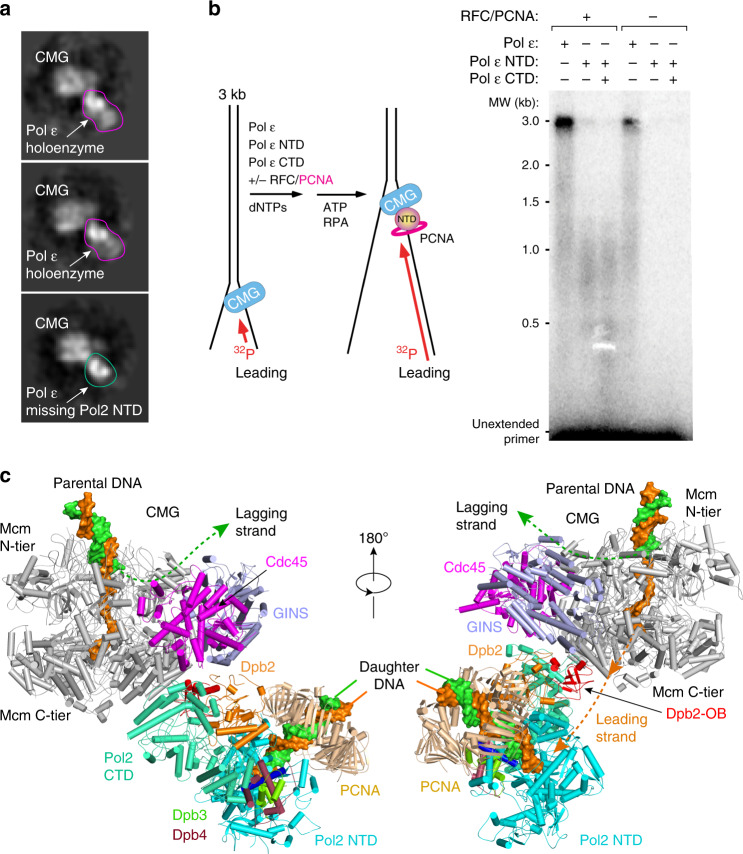
Atomic model of the leading strand replisome. **a** 2D class averages of cryo-EM images of purified native Pol ε in complex with recombinant CMG. **b** A 3-kb forked DNA, primed with a 5′-^32^P-DNA oligonucleotide, is replicated in the presence of CMG, RPA, PCNA, and RFC, using either Pol ε holoenzyme (lane 1), catalytic Pol2 NTD (lane 2), or catalytic Pol2 NTD plus Pol2 CTD (lane 3). Lanes 4–6 are the same as lanes 1–3, except in the absence of PCNA and RFC. **c** A model showing how the CMG helicase is coupled with the holoenzyme Pol ε in which the Dpb2 OB domain likely bridges the leading strand DNA path from CMG to the Pol ε catalytic entry. The leading strand path is drawn as an orange dashed line with two arrowheads indicating the ssDNA moving direction.

The fact that our Pol ε structure is in agreement with the solution cross-linking data of the Pol ε in complex with CMG suggests that Pol ε can take on the ordered rigid state in the leading strand replisome in the presence of CMG helicase. We asked if the structure of endogenous Pol ε that is purified in complex with expressed recombinant CMG^8^ would be able to display both flexible and rigid states as observed in the isolated recombinant Pol ε. Cryo-EM of the native Pol ε bound to recombinant CMG revealed that Pol ε indeed had both the rigid and flexible states in 2D class averages, but it remains possible that the cryogenic process of EM grid preparation might explain or contribute to the flexible form (Fig. 4a). In addition to helicase and polymerases, sliding clamps like PCNA are also an integral part of a replisome by encircling duplex DNA while tethering their respective polymerase during replication^32–34^. While we presume PCNA binds the NTD of Pol2, there is no data that we are aware of to test this conjecture. Thus, we cloned and expressed the Pol2 catalytic NTD and tested it in a replisome assay using CMG, RPA, ±PCNA/RFC. In this assay, a 3 kb duplex is ligated to a synthetic replication fork and leading strand replication is primed with a 5′-^32^P oligonucleotide (Fig. 4b). The results show that PCNA is required to observe replication activity by the Pol2 catalytic NTD (compare lanes 2 and 5). Addition of Pol ε CTD did not result in further stimulation (compare lanes 2 and 3). Our result using the forked DNA as a substrate is in agreement with earlier reports using polydA-dT^35^ with the yeast Pol2 NTD and using primed M13 ssDNA with human p261 NTD^30^. Hence, we conclude that the Pol2 NTD directly interacts with PCNA.

The structure of Pol ε holoenzyme, together with our knowledge of CMG helicase in complex with Pol ε, and that PCNA binds to the catalytic domain of Pol2, enabled us to build a pseudo atomic model of the leading strand replisome composed of CMG–forked DNA–Pol ε–primer/template–PCNA (Fig. 4c and Supplementary Movie 2). We first docked the structures of CMG–forked DNA^36^ and our Pol ε structure into the 4.5–7 Å cryo-EM 3D map of CMG–DNA–Pol ε_ΔPol2-NTD^11^. We found that only minor rigid-body motions in the Pol2 CTD were required to fit our Pol ε structure into the lower resolution 3D map, and thus tentatively conclude that Pol ε binding to CMG causes no significant distortion of either the CMG helicase or the rigid form of the Pol ε holoenzyme. We next derived the template/primer position in Pol2 NTD by superimposing the Pol2 NTD region of the published Pol2_NTD–P/T structure^22^ with our apo holoenzyme. The binding position of the PCNA was then derived by superimposing the catalytic domain of the human Pol δ–DNA-FEN1–PCNA structure^37^ with the Pol2 NTD of the Pol ε holoenzyme; this was feasible because the structural fold of the catalytic domain of the three eukaryotic replicative polymerases are highly conserved.

The Pol2 PIP motif (1193–1201^35^) is within the first half of the NTD–CTD linker (aa 1187–1270), thus is disordered in the Pol ε holoenzyme structure. However, the last helix and a short loop (1177–1186) in the Pol2 NTD, which immediately precedes the PIP motif, is located right above the PIP binding site and the interdomain connection loop of PCNA (Supplementary Fig. S9a–c). Therefore, the PIP motif is positioned to interact with PCNA. Although there is no PIP motif in the Pol2 P domain, PCNA can tilt up and down with respect to Pol δ to narrow the gap between the P domain and PCNA^37^ making it possible that the Pol2 P domain may interact with PCNA, just like FEN1 does in the Pol δ–PCNA–FEN1 complex^37^. Further, a Dpb2 helix and the connecting loop (aa 378–399) and a Pol2 NTD loop (aa 1125–1139) are also close to the PCNA NTD–CTD junction; they may interact with PCNA as well (Supplementary Fig. S9b, c). In our model, the primer end is bound by the Pol2 NTD which is stabilized by PCNA. This may raise the question of how an RFC clamp loader or Pol α polymerase-primase gain access to the primer/template DNA. We suggest that PCNA is loaded by RFC on the initial primer before Pol ε binding; then the primer is extended by Pol δ or directly taken over by Pol ε. Furthermore, the Pol2 NTD is flexible in ~80% of the particles in the cryo-EM dataset, suggesting the catalytic domain periodically vacates the P/T junction to allow binding of RFC, Pol α, or a translesion DNA polymerase. This is consistent with the demonstration that neither Pol δ nor Pol ε prevents PCNA clamp loading^38^.

**Fig. 5:**
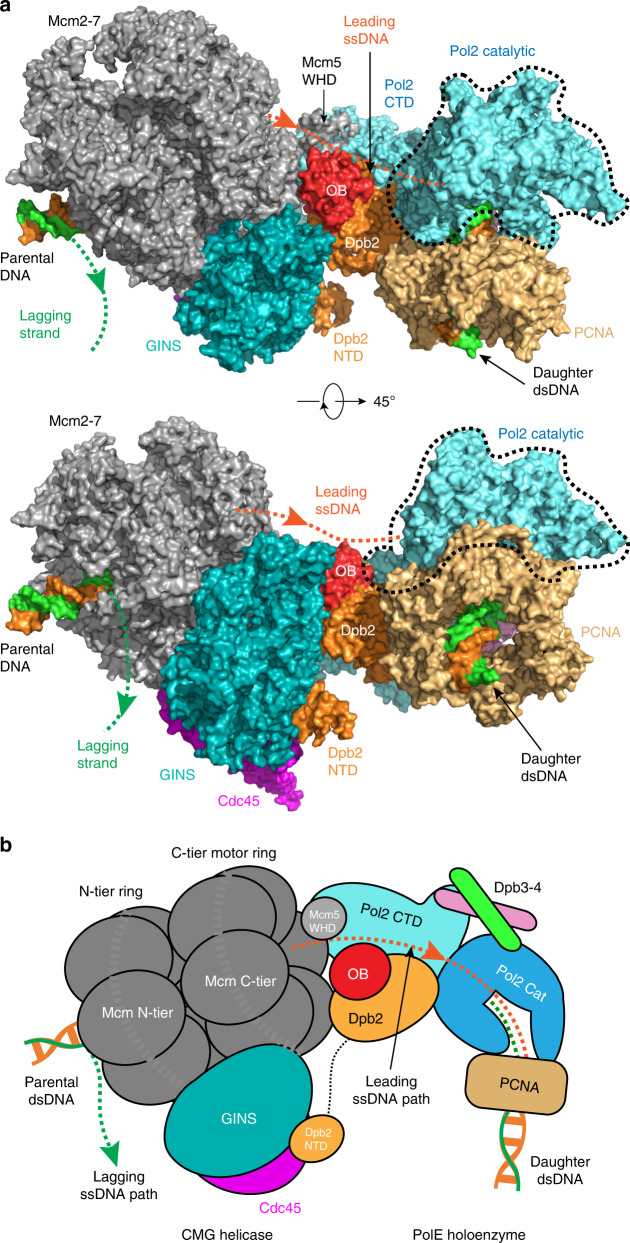
Leading ssDNA path from the CMG helicase to the Pol ε. **a** Top and side views of the surface presentation of the atomic model of the leading strand replisome, centering around the Dpb2 OB domain. **b** A sketch illustrating the midway location of Dpb2 in the shortest path of the leading strand DNA from CMG to the entrance of the Pol ε catalytic site.

It has been unknown how the leading strand DNA exiting the CMG helicase is directed to the Pol ε catalytic site and how the nascent daughter DNA exits the Pol ε in the context of a replisome. This information is essential for understanding how the leading strand is replicated. In our model of the leading strand replisome, we found that the nascent daughter duplex is extruded from Pol ε–PCNA approximately perpendicular to the parental duplex entering the CMG (Fig. 4c). This perpendicular arrangement of the parent and daughter DNA is reminiscent of the T7 replisome^39^ although the phage and eukaryotic replication systems are evolutionarily unrelated^40^. The leading strand DNA emerging from the C-tier motor ring of CMG travels apparently unobstructed over a distance of ~100 Å before reaching the Pol2 polymerase active site through an opening (Fig. 5a, b). Interestingly, the Mcm5 WHD was observed to bind the Pol2 CTD in-line with the leading strand ssDNA exit in the cryo-EM map of CMG–Pol2 CTD–Dpb2^15^. Dpb2 is the only essential subunit beyond the catalytic Pol2 in the Pol ε holoenzyme, but its function has been obscure. The Dpb2 NTD is disordered in the holoenzyme structure but was found to bind between GINS and Cdc45 of CMG helicase (Fig. 5a, b)^15^. The OB fold (oligosaccharyl/oligonucleotide binding domain) such as those found in SSB and RPA is known to bind ssDNA. The Dpb2 OB domain is positioned midway in the journey of the leading strand DNA from the helicase exit to the entry of the polymerase catalytic site. Therefore, we suggest that both the Mcm5 WHD and the Dpb2 OB may play roles in guiding the leading strand. The suggested leading ssDNA path is the shortest path from the CMG exit to the Pol ε entry site and is partially positively charged, suitable for guiding the negatively charged ssDNA (Supplementary Fig. 10). Based on the replisome architecture, we suggest that the Dpb2 OB domain bridges the leading strand DNA path between the helicase and the polymerase. We note that the 100 Å distance from CMG to Pol ε, equivalent to a 16-base stretch of ssDNA, would not be fully exposed to the solvent, as it would be partially shielded/protected by the flexible components of the replisome along the DNA path, including the C-terminal winged helix domains of the Mcm subunits and the NTD of the Dpb2.

Previous studies document a flexible form of Pol ε^10,15,26^, and a flexible form of Pol ε is also noted here, but whether this is an artifact of cryo-grid preparation or whether a flexible form of Pol ε exists in the cell is unknown. It is possible that a flexible form of Pol ε, if it exists, serves a physiological role. An obvious possibility is that a flexible catalytic domain of Pol ε may provide access to the 3′ terminus for another DNA polymerase, such as a TLS polymerase upon encounter with a DNA lesion. It is also tempting to speculate that a flexible form of Pol ε may facilitate transfer of parental nucleosomes to the leading strand for epigenetic inheritance. Cellular studies show that Dpb3 and Dpb4 are required for epigenetic inheritance, as deletion of the gene encoding them result in deficiency in transfer of parental epigenetic information to the leading strand^20,21,41,42^. Interestingly, Dpb3–4 are demonstrated to bind the H3–H4 tetramer^42^. We note that if the binding between Dpb3–4 and H3–H4 mimics the interface between H2A–H2B and H3–H4 in the histone octamer, the rigid Pol ε holoenzyme structure determined here is incompatible with Dpb3–4 binding to H3–H4 because the Pol2 mooring helix gets in the way preventing such interaction. Perhaps a flexible form of Pol ε might possibly facilitate H3–H4 binding by Dpb3–4. The role of Dpb3–Dpb4 in epigenetics is an important issue that requires further study.

In summary, we have solved the structure of the eukaryotic Pol ε holoenzyme, revealing how Dpb3–4 is anchored in the middle of the two-lobed enzyme complex by the mooring helix which is part of the long linker between the active and inactive polymerase modules of Pol2. The structure has also enabled atomic modeling of the entire leading strand replisome, revealing a likely path for the leading strand DNA from the helicase to the polymerase catalytic site, through the PCNA clamp, and the likely function of the Dpb2 OB domain in bridging the helicase and polymerase in directing the leading strand through the replisome.

## Methods

### Proteins and nucleic acids

Pol ε holoenzyme was purified integrating the genes for its subunits into yeast under control of the Gal1/10 promotor. Yeast cells were then grown in YP media at 30 °C, and induced upon reaching an OD_600_ of 0.7 by addition of 20 g/L galactose followed by continued growth for 6 h. Pol ε holoenzyme was then purified similarly to a previously method^43^. Briefly, cells were lysed using a SPEX cryogenic grinding mill (6970 EFM), and cell debris was removed by centrifugation (19,000 r.p.m. in a SS-34 rotor for 1 h at 4 °C). The supernatant was applied to a 1 ml Anti-Flag antibody resin (Sigma), followed by elution using 0.15 mg/ml 3XFLAG peptide (EZBiolab, Carmel, Indiana, USA). Peak fractions were then pooled and further purified on a MonoS column. Peak fractions containing Pol ε holoenzyme were pooled and dialyzed against 50 mM Hepes pH 7.5, 50 mM KGlu, 200 mM KAcetate, 1 mM DTT, 4 mM MgCl_2_, aliquoted, snap frozen in liquid nitrogen and stored at −80 °C. CMG was purified by integrating each of the 11 genes encoding the CMG subunits into yeast, similar to a previous protocol^8^. Briefly, yeast cells were grown in YP media at 30 °C and induced at an OD_600_ of 0.7 by addition of 20 g/L galactose and incubated a further 6 h at 30 °C. CMG was then purified similar to Pol ε holoenzyme through the FLAG antibody affinity column step. Peak fractions were then pooled and further purified on a 1 ml HisTrap HP column (GE Healthcare) and eluted with a 7.5 ml linear gradient of 5–750 mM imidazole. Peak fractions were pooled and dialyzed against 50 mM Hepes pH 7.5, 50 mM KGlu, 200 mM KAcetate, 1 mM DTT, 4 mM MgCl_2_, aliquoted, snap frozen in liquid nitrogen, and stored at −80 °C

### DNA replication assay

Experiments used a 2.7 kb forked DNA substrate having a synthetic forked DNA ligated to linearized pUC19 DNA, followed by gel filtration to remove excess forked DNA as described^38^. The DNA template was primed using a 5′ ^32^P-37mer oligonucleotide as described^38^. Reactions (25 μL final) contained 1.25 nM linear forked template in 25 mM Tris-OAc pH 7.5, 5% glycerol, 40 μg/ml BSA, 5 mM DTT, 10 mM Mg-OAc, 50 mM K glutamate, 0.1 mM EDTA, and 120 μM of dNTPs. Replication assays were performed by first incubating 20 nM CMG with 1.25 nM linear forked template for 10 min on ice followed by adding 5 nM RFC, 25 nM PCNA, and 40 nM of either Pol ε, Pol ε NTD, or Pol ε CTD for an additional 5 min at 30 °C. Reactions were initiated with 5 mM ATP and 600 nM RPA and allowed to run 20 min at 30 °C. Reactions were quenched upon adding 0.5% SDS, 20 mM EDTA (final) and analyzed in a 1.2% alkaline agarose gel. Gels were dried, exposed to phosphorimager screens, and imaged using a Typhoon FLA 9500 PhosphorImager (GE Healthcare).

### Cryo-EM

To prepare EM grids of Pol ε samples, 3 μl of Pol ε sample was applied, at a final concentration of ~1.6 mg/ml, to C-flat 1.2/1/3 holey carbon grids, treated by glow-discharge before use. Grids were then incubated for 10 s at 6 °C and 90% humidity, blotted for 3 s and plunged into liquid ethane using a Thermo Fisher Vitrobot IV. Grids were loaded into a Titian Krios electron microscope and images at 300 kV were collected automatically using low-dose mode at a magnification of ×130,000 and a pixel size of 1.029 Å per pixel. A Gatan K2 summit direct electron detector was used for image recording with a defocus range from −1.5 to −2.5 μm under super-resolution mode. The dose rate was 10 electrons per Å^2^ per second and total exposure time was 6 s. The total dose was divided into 30-frame movies and each frame was exposed for 0.2 s. For cryo-EM of the CMG-Pol ε complex containing native Pol ε and recombinant CMG, the sample concentration was 1.1 mg/ml. The cryo-EM data was collected in an Arctica Talos electron microscope operated at 200 kV with a Gatan K2 summit direct electron detector. The dose rate was 10 electrons per Å^2^ per second and total exposure time was 6 s. The total dose was divided into 30-frame movies and each frame was exposed for 0.2 s.

### Image processing and 3D reconstruction

Over 6000 raw movie micrographs were collected. Firstly, all the movie frames were aligned and superimposed by Motioncorr2^44^. Contrast transfer function parameters of each aligned micrograph were calculated with CTFFIND4^45^. All the subsequent steps, including particle autopicking, 2D classification, 3D classification, 3D refinement, postprocessing, were performed in Relion-2.0^46^. We manually picked about 2000 particles from different views to generate several 2D averages which were subsequently used as a template for automatic particle picking. Automatic particle picking was then performed for the whole dataset. About 1,033,695 particles were initially picked. These were then sorted according to the similarity to the 2D reference; the bottom 10% particles that had very low *z*-scores were deleted from the particle pool. The 2D classification of all the remaining particles was performed and particles in bad classes (i.e. only Pol2 CTD–Dpb2) were removed. Overall 749,195 good particles (Pol ε holoenzyme structure was observed) were kept for the following 3D classification. We derived five 3D models from the dataset, and refinement of the best model led to the final 3D map with an average resolution of 3.5 Å. The resolution estimation was based on gold-standard Fourier shell correlation calculation to avoid over-fitting and the reported resolution was based on the FSC = 0.143 criterion. The 3D map was corrected for the modulation transfer function of the detector and sharpened by applying a negative B-factor. Local resolution was estimated using ResMap^47^. These steps are illustrated and summarized in Supplementary Figs. 1–3.

### Atomic modeling and validation

Models of all *S. cerevisiae* Pol ε subunits were directly extracted from the crystal and cryo-EM structure of the yeast Pol2 NTD (PDB ID 5U8S), Pol2 CTD with Dpb2 (PDB ID 6HV8), and Dpb3–Dpb4 (PDB ID 5Y26). DNA sequence was randomly assigned in the model. These models were rigid-body fitted into the 3D density map with COOT^48^ and Chimera^49^. The entire Pol ε models were firstly refined by rigid-body refinement of individual chains using the PHENIX program, and subsequently adjusted manually in COOT guided by residues with bulky side chains like Arg, Phe, Tyr, and Trp. The electron densities at the metal binding sites in the C-terminal CysA and CysB of Pol2 CTD were weak, particularly at the CysB site. We modeled a Zn^2+^ in the CysA site (aa 2108–2130) but did not model a ligand in the CysB site. The model was then refined in real space by phenix.real_space_refine and in reciprocal space by PHENIX with the application of secondary structure and stereochemical constraints^50^. The structure factors (including phases) were calculated by Fourier transform of the experimental density map with the program Phenix.map_to_structure_factors. The final models were validated using MolProbity^51^. Structural figures were prepared in Chimera and Pymol (https://www.pymol.org).

Modeling of the leading strand replisome involved the following four steps using the published 5 Å cryo-EM 3D map of the yeast CMG–Pol2_CTD–Dpb2 (EMD-0288): (1) Docking our 3.9-Å resolution cryo-EM structure of CMG–forked DNA (PDB ID 6U0M) into the 5 Å 3D map of CMG–Pol2_CTD–Dpb2; (2) docking our 3.5 Å resolution cryo-EM structure of Pol ε holoenzyme determined in this report into the 5 Å 3D map of CMG–Pol2_CTD–Dpb2; (3) Superimposing the 2.2 Å crystal structure of the yeast Pol2 NTD–DNA template/primer (PDB ID 4M8O) into the Pol2 NTD region of our Pol ε holoenzyme to generate the DNA T/P bound form of the holoenzyme; (4) Superimposing the polymerase catalytic domain of our Pol ε holoenzyme with that of the 4 Å cryo-EM structure of human Pol δ–FEN1–PCNA-structure (PDB ID 6TNZ) to derive the position of PCNA and thus the model of PCNA- and DNA P/T-bound to Pol ε.

### Reporting summary

Further information on research design is available in the Nature Research Reporting Summary linked to this article.

## Supplementary information

Supplementary Information

Reporting Summary

Description of Additional Supplementary Files

Supplementary Movie 1

Supplementary Movie 2

## Data Availability

The 3D cryo-EM map of the *S. cerevisiae* polymerase ε holoenzyme at 3.5 Å resolution has been deposited in the Electron Microscopy Data Bank under accession code EMD-21701. The corresponding atomic model has been deposited in the Protein Data Bank under accession code PDB 6WJV.
