# Supplementary material for: Structure of the polymerase ε holoenzyme and atomic model of the leading strand replisome
Source: Nat Commun. 2020 Jun 22;11:3156. doi: 10.1038/s41467-020-16910-5 (PMC7308368; doi:10.1038/s41467-020-16910-5)
Supplement: Supplementary file 1 — Supplementary Information [file 41467_2020_16910_MOESM1_ESM.pdf]

Supplementary Information for:

Structure of the polymerase ε holoenzyme and atomic model of the leading strand replisome

By Yuan et al.

Supplementary Table 1. Cryo-EM 3D reconstruction and atomic model validation

	Pol ε holoenzyme
**Data collection and processing**	
Magnification	130,000
Voltage (kV)	300
Electron dose (e^-/Å^2)	60
Under-focus range (μm)	1.5 – 2.5
Pixel size (Å)	1.029
Symmetry imposed	C1
Initial particle images (no.)	1,033,695
Final particle images (no.)	187,298
Map resolution (Å)	3.5
FSC threshold	0.143
Map resolution range (Å)	3.5 – 5.0
**Refinement**	
Initial model used (PDB code)	5U8S, 6HV8, 5Y26
Map sharpening B factor (Å^2)	-122
Model composition	
Non-hydrogen atoms	20,523
Protein and DNA residues	2,580
Ligands	0
R.m.s. deviations	
Bond lengths (Å)	0.009
Bond angels (°)	1.22
Validation	
MolProbity score	2.17
Clashscore	10.58
Poor rotamers (%)	0.68
Ramachandran plot	
Favored (%)	86.45
Allowed (%)	13.51
Disallowed (%)	0.04

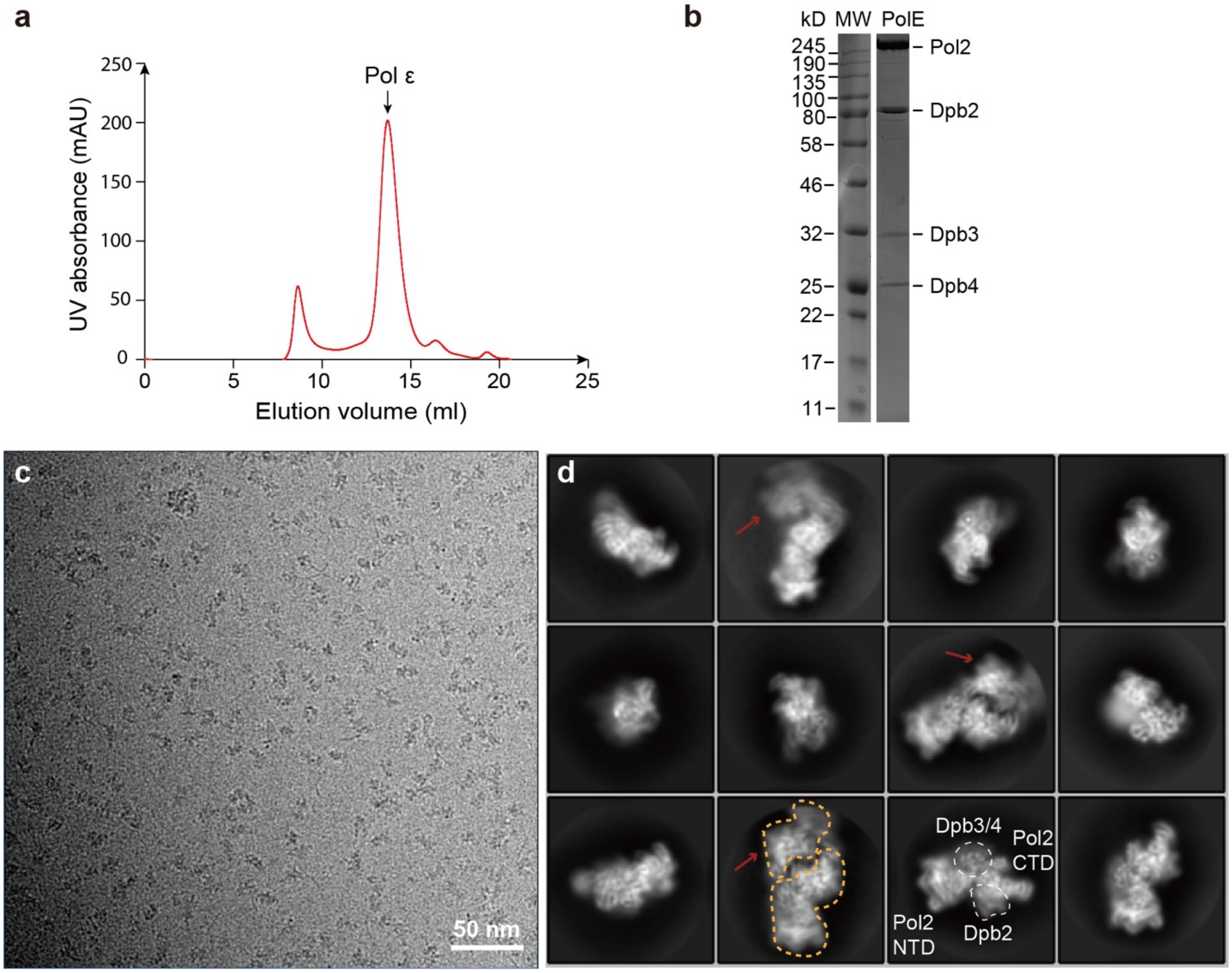

Supplementary Figure 1. Cryo-EM of Pol ε bound to primed DNA. a) UV profile of Pol ε elution from a gel filtration column. **b**) Coomassie Blue stained SDS-PAGE of purified pol ε. **c**) A representative raw micrograph. **d**) 2D class averages showing the presence of both monomeric and dimeric Pol ε particles in solution.

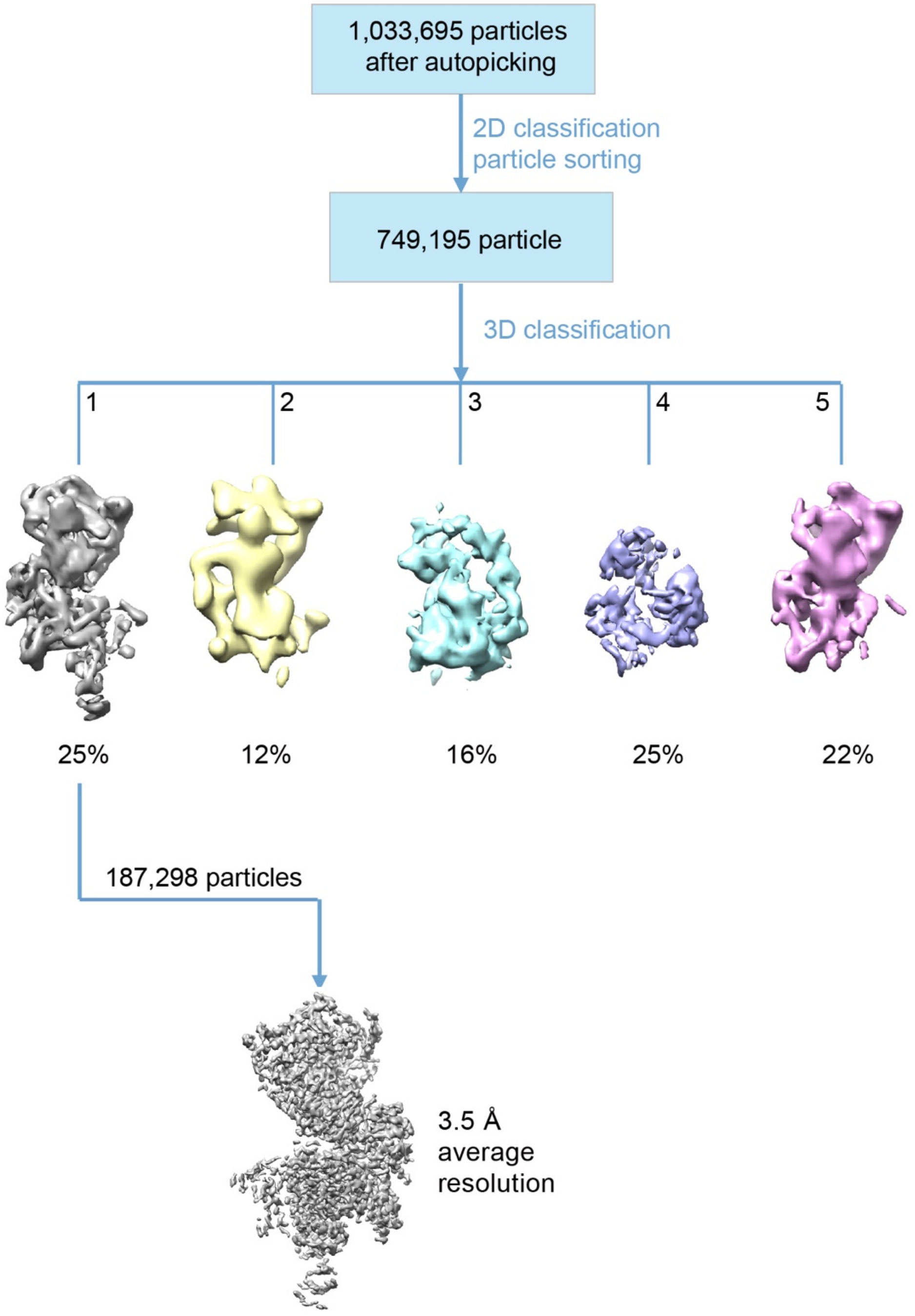

Supplementary Figure 2. 3D classification and refinement leading to the final 3.5 Å 3D map.

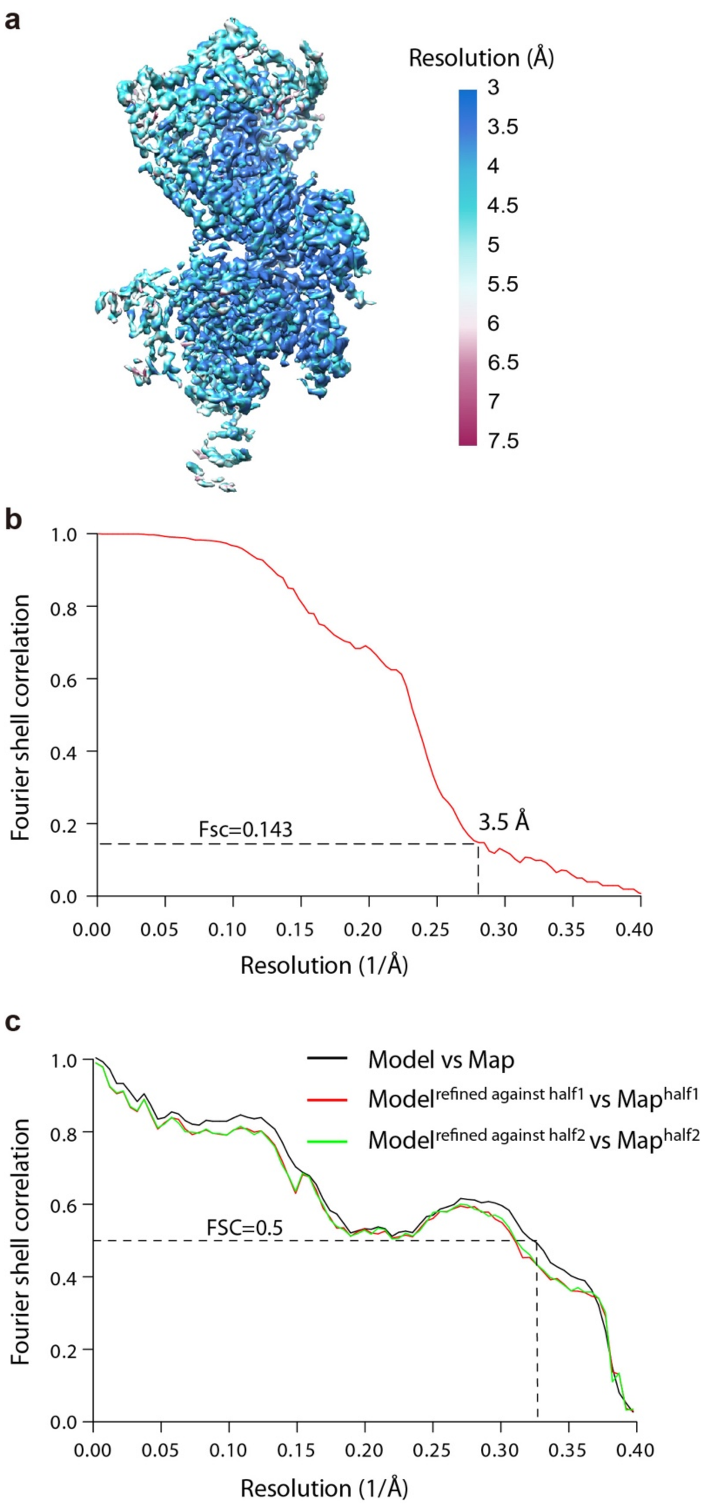

Supplementary Figure 3. Resolution estimation of the 3D map. a) Local resolution map. **b**) Gold standard Fourier shell correlation of two half maps. **c**) Fourier shell correlations between model and map and model with two half maps.

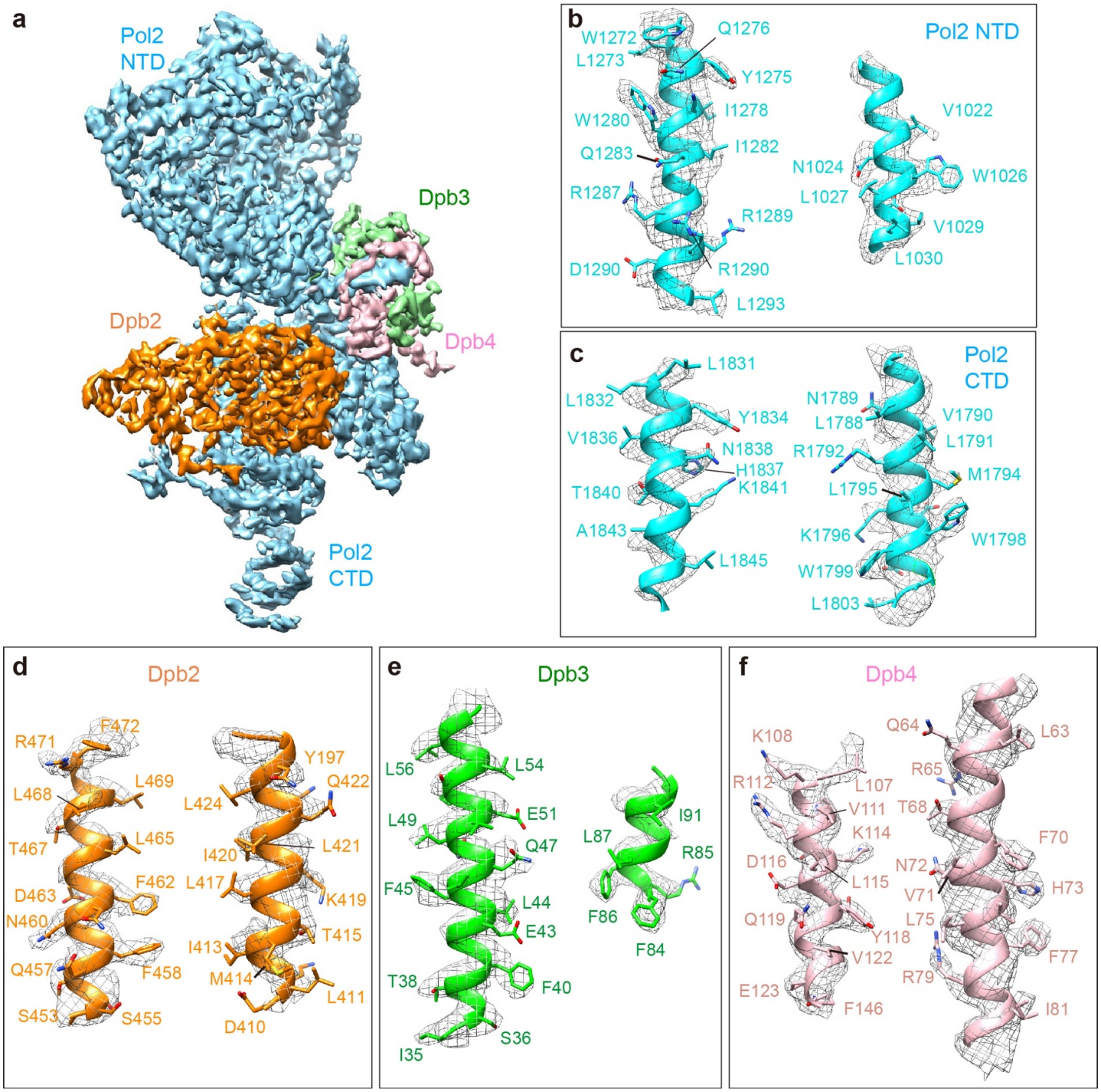

Supplementary Figure 4. Density details from selected regions in each of the four subunits. a) Surface rendering of the cryo-EM 3D map of the Pol ε holoenzyme, with subunits individually colored and labeled. **b-f**) Selected helical regions of Pol2 NTD, Pol2 CTD, Dpb2, Dpb3 and Dpb4 showing the densities of large side chains.

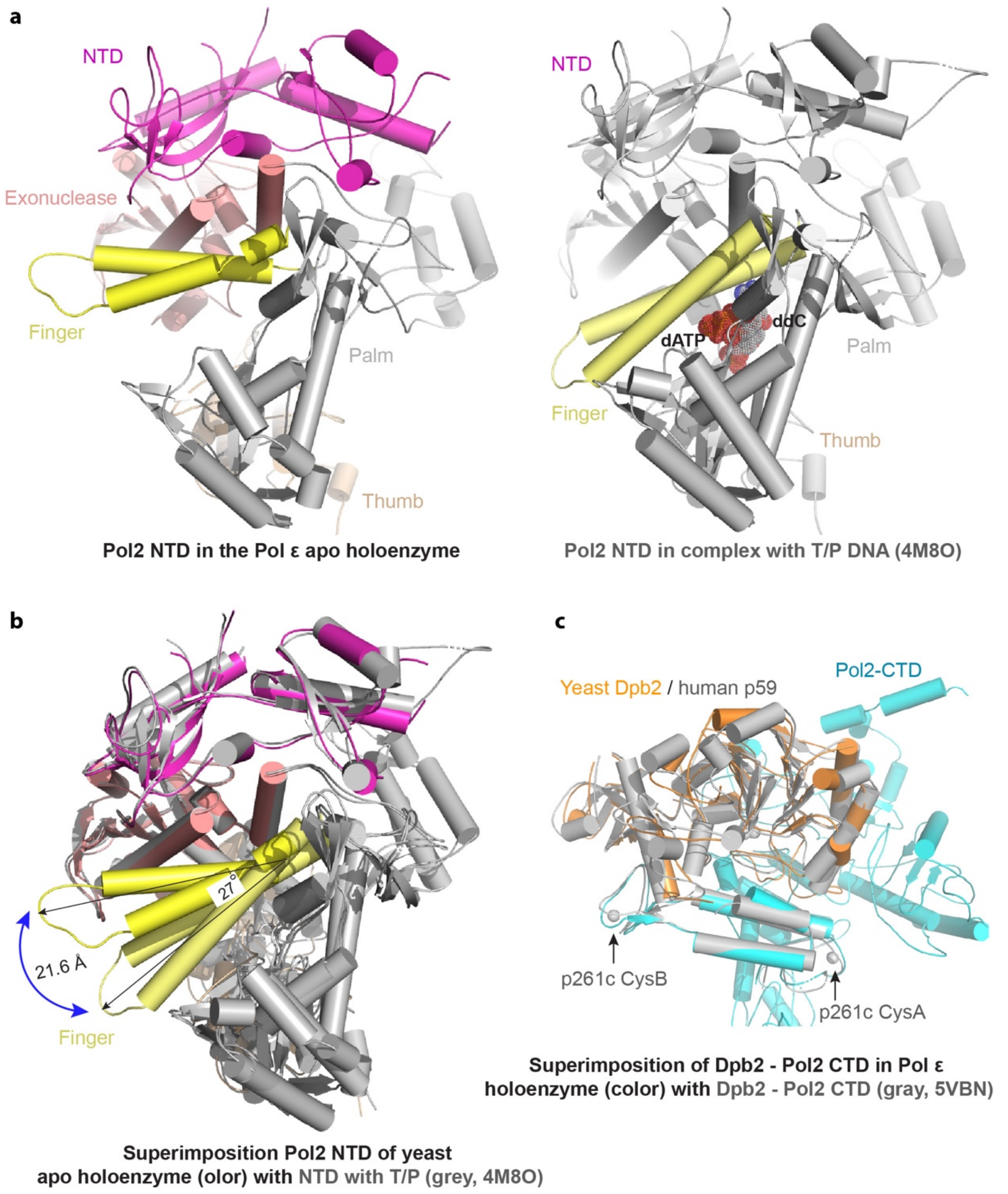

Supplementary Figure 5. Comparison of the S. c. Pol ε holoenzyme with two published partial crystal structures. a) Comparison of the catalytic Pol2 NTD structure in the apo holoenzyme (left) and in the T/P DNA bound crystal structure (right, PDB ID 4M8O)). The T/P is omitted and only the 3' terminal and the incoming nucleotides are shown in spheres. **b**) Superimposition of the structures reveals a 27º tilt of the finger domain to clamp down and bind the T/P DNA. **c**) Superimposition of the CMG helicase-binding region, the Dpb2-Pol2_CTD of the yeast holoenzyme (color) with the crystal structure of human p59 in complex with the p261 C-terminal Zinc-binding fragment (p261c CysA and CysB) (grey, PDB ID 5VBN).

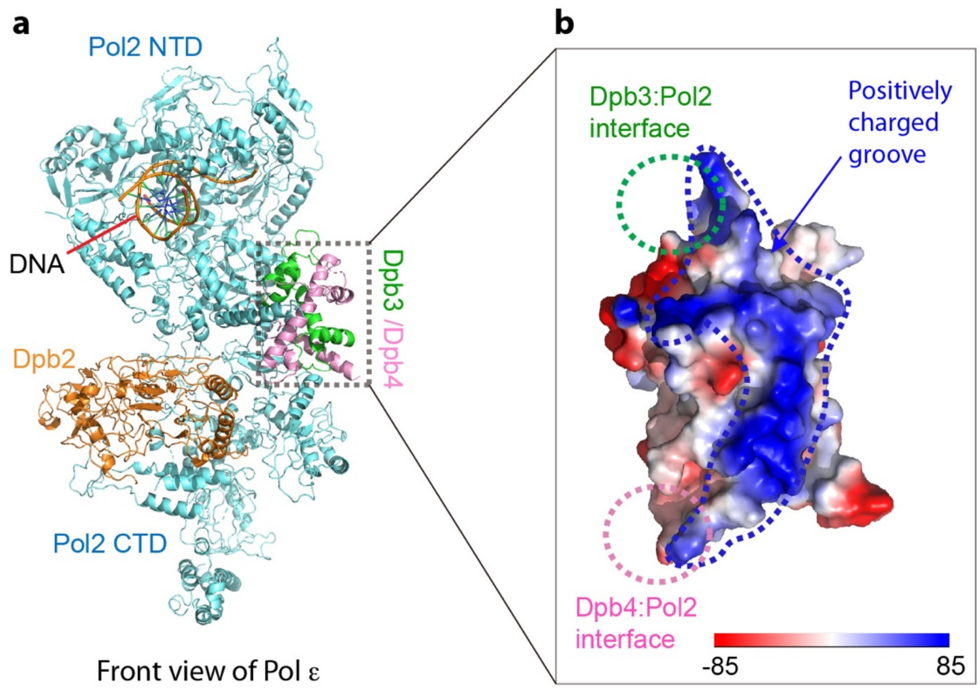

Supplementary Figure 6. A potential DNA binding site on Dpb3–4. **a**) Atomic model of the Pol ε in a front side view with each subunit individually colored. **b**) A surface charge plot of Dpb3–4 as viewed from the same direction as in panel a.

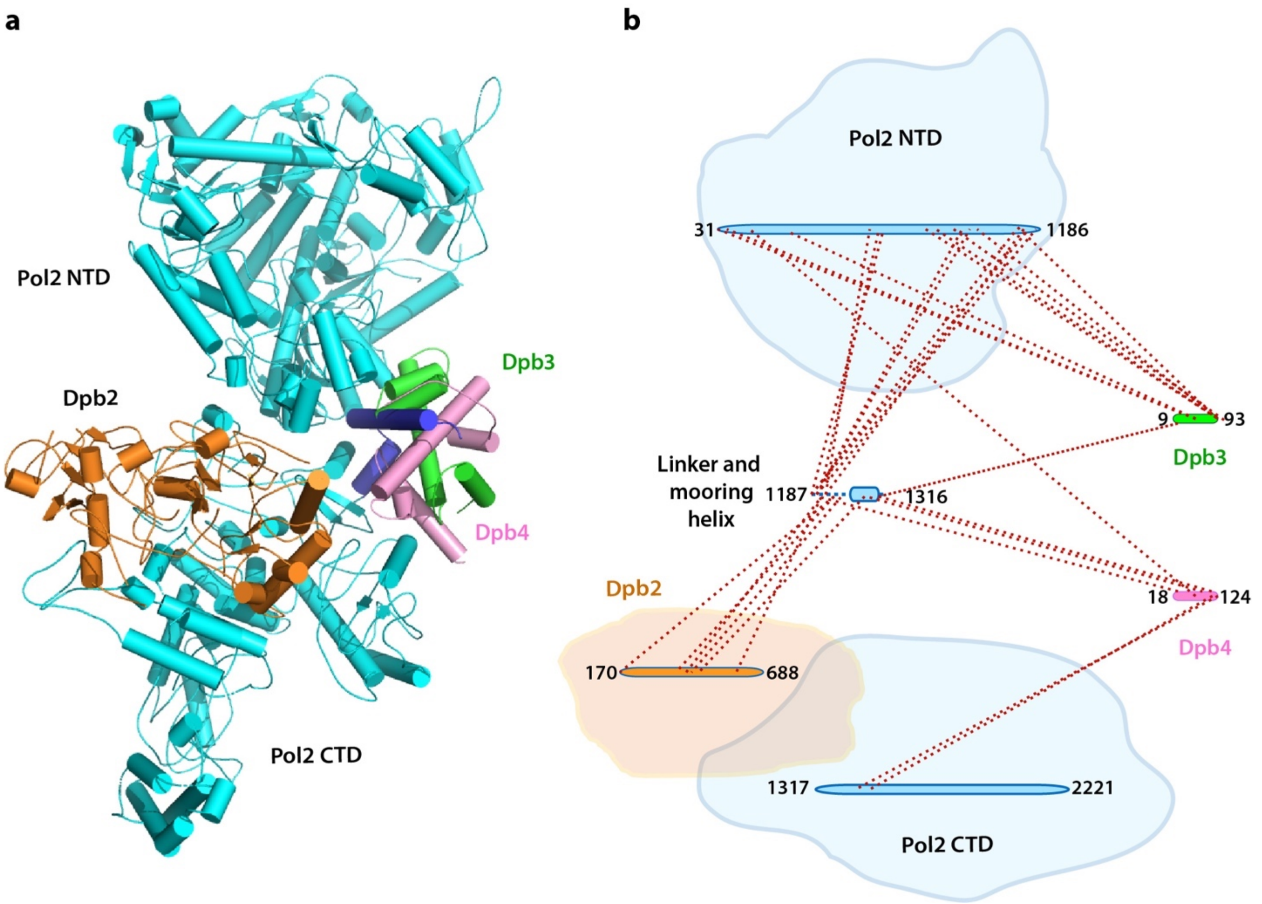

Supplementary Figure 7. CX-MS cross-link data mapped onto the rigid state structure of Pol ε holoenzyme. a) Cartoon view of the cryo-EM structure of Pol ε. **b)** A sketch showing the inter-subunit cross-linking reported in our previous study [10].

a

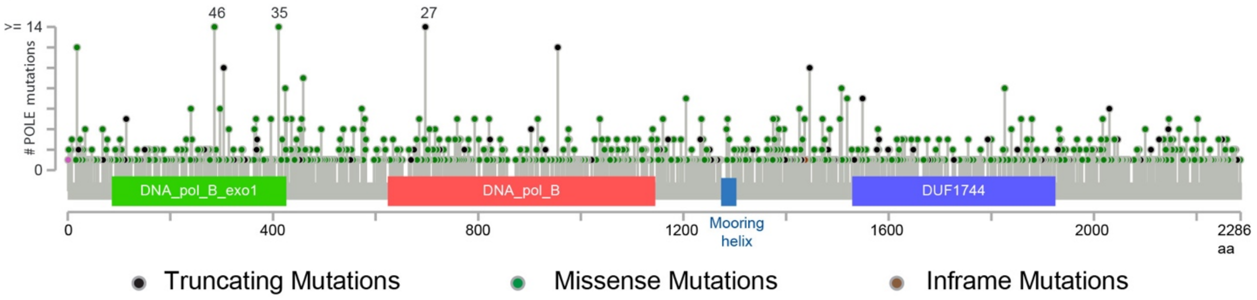

b

Human Pol2 mutations	Corresponding S.c. Pol2 residue	Mutation type	Associated diseases
R1284L	R1285	Missense	Lung Adenocarcinoma
R1284Q	R1285	Missense	Mature B-cell Neoplasms
R1284W	R1285	Missense	Lung Adenocarcinoma
Q1285K	D1286	Missense	Breast Invasive Ductal Carcinoma
R1286C	R1287	Missense	Upper Tract Urothelial Carcinoma
R1286H	R1287	Missense	Gallbladder Cancer

Supplementary Figure 8. Human Pol2 mutations identified in Cancer Genomics Database (cBioPortal). **a**) Mutations in the entire catalytic Pol2 subunit. **b**) Mutations in the ordered mooring helix in the Pol2 NTD-CTD linker.

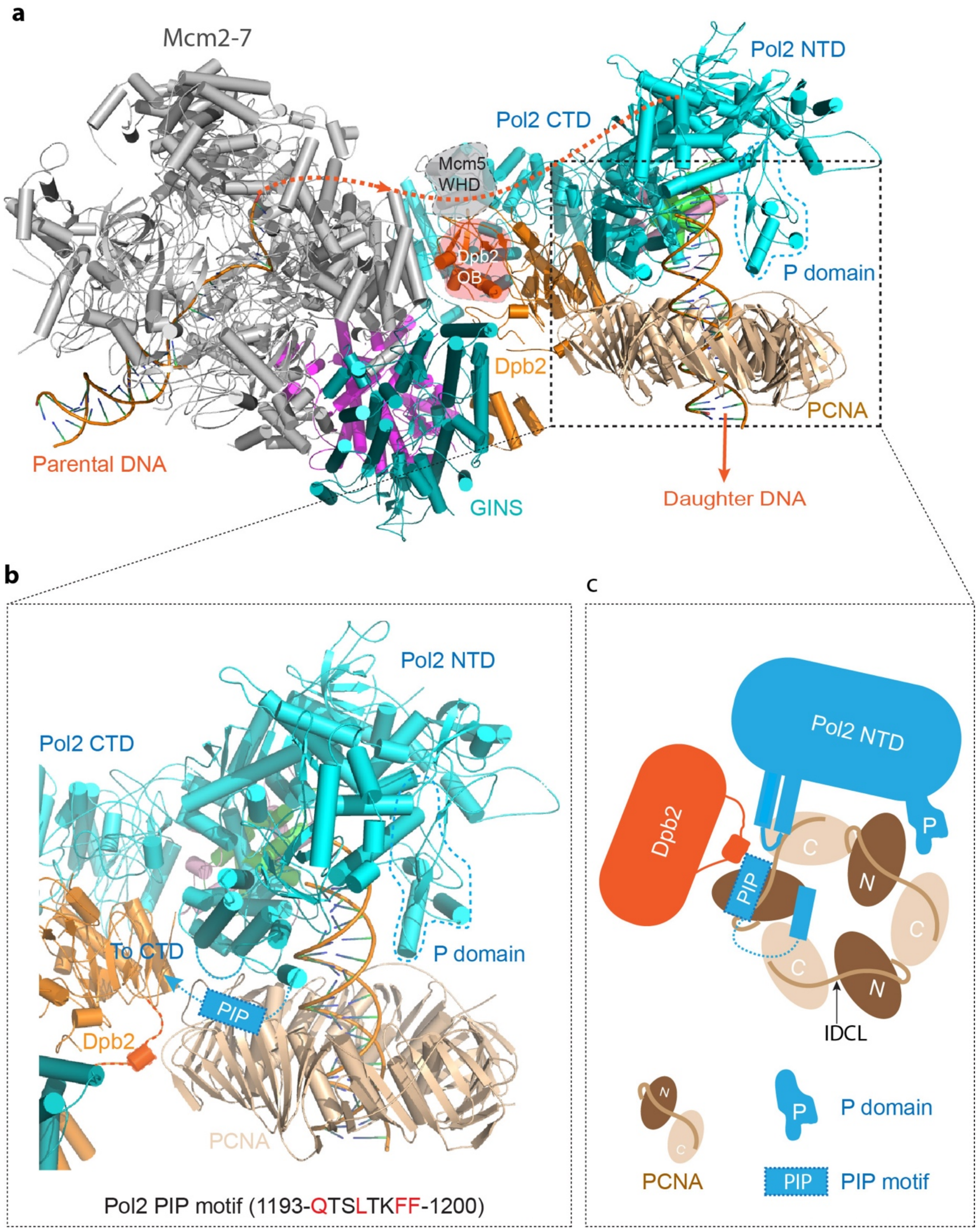

Supplementary Figure 9. Potential interactions between Pol ε and PCNA. a) Atomic model of the CMG-Pol ε-T/P DNA-PCNA. **b)** Enlarged view of the boxed region in panel a. **c**) A sketch of panel b, showing the possible interactions between Pol2 PIP and P domain as well as a loop region in Dpb2 with PCNA ring.

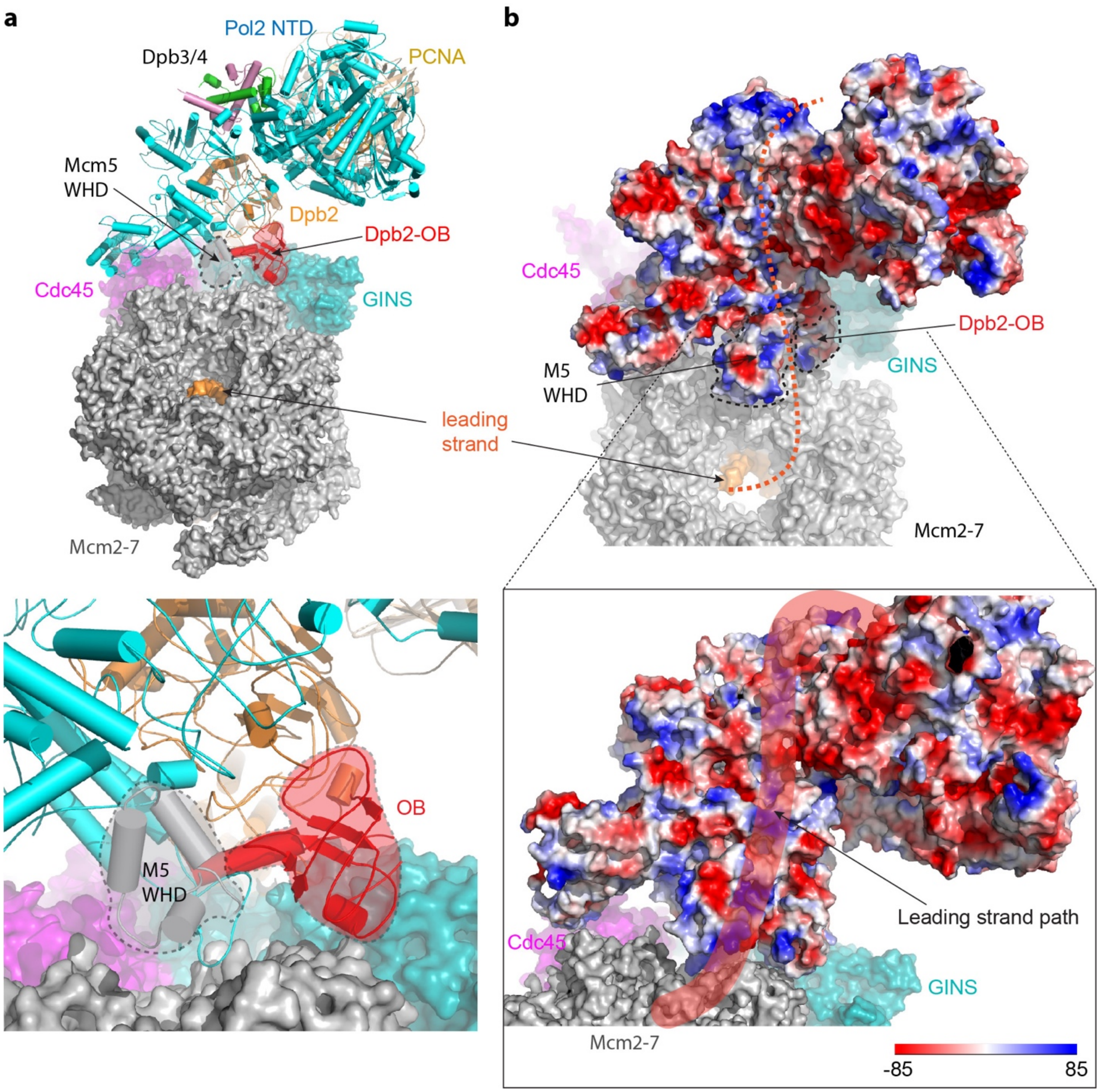

Supplementary Figure 10. Details of the leading ssDNA path from CMG helicase to the Pol2 catalytic site. a) Atomic model of the leading strand replisome with Pol ε in cartoon and CMG in surface plot (top) and an enlarged view around the Dpb2 OB domain region (bottom). **b**) The Pol ε is shown as surface charge in the atomic model of the leading strand replisome (top). The bottom panel is an enlarged view around the Dpb2 OB domain.
