# Supplementary material for: Structure of the polymerase ε holoenzyme and atomic model of the leading strand replisome
Source: Nat Commun. 2020 Jun 22;11:3156. doi: 10.1038/s41467-020-16910-5 (PMC7308368; doi:10.1038/s41467-020-16910-5)
Supplement: Supplementary file 3 — Description of Additional Supplementary Files [file 41467_2020_16910_MOESM3_ESM.docx]

Description of Additional Supplementary Files

File Name: Supplementary Movie 1

Description: 3D structure of the yeast polymerase epsilon holoenzyme. The movie starts with a 360° rotation of the 3D map around a vertical axis, transition to a cartoon view of the atomic model, then zoom into the middle region where the mooring helix braces Dpb3-Dpb4.

File Name: Supplementary Movie 2

Description: Atomic modeling of the leading strand replisome. A 180° rotation around a vertical axis of the model in cartoon presentation, with a zoom into the leading strand ssDNA path from the helicase exit to the polymerase entry, continued with another 180° rotation to complete the full turn.
